# Morphological and Transcriptomic Analysis Reveals the Osmoadaptive Response of Endophytic Fungus *Aspergillus montevidensis* ZYD4 to High Salt Stress

**DOI:** 10.3389/fmicb.2017.01789

**Published:** 2017-09-21

**Authors:** Kai-Hui Liu, Xiao-Wei Ding, Manik Prabhu Narsing Rao, Bo Zhang, Yong-Gui Zhang, Fei-Hu Liu, Bing-Bing Liu, Min Xiao, Wen-Jun Li

**Affiliations:** ^1^School of Biological Science and Engineering, Shaanxi University of Technology Hanzhong, China; ^2^State Key Laboratory of Biocontrol and Guangdong Provincial Key Laboratory of Plant Resources, School of Life Sciences, Sun Yat-Sen University Guangzhou, China; ^3^School of Life Sciences, Yunnan University Kunming, China; ^4^Key Laboratory of Biogeography and Bioresource in Arid Land, Xinjiang Institute of Ecology and Geography, Chinese Academy of Sciences Ürűmqi, China

**Keywords:** transcriptome, halophilic endophytic fungi, *Aspergillus montevidensis*, high-salt stress, osmoadaptive mechanisms

## Abstract

Halophilic fungi have evolved unique osmoadaptive strategies, enabling them to thrive in hypersaline habitats. Here, we conduct morphological and transcriptomic response of endophytic fungus (*Aspergillus montevidensis* ZYD4) in both the presence and absence of salt stress. Under salt stress, the colony morphology of the *A. montevidensis* ZYD4 changed drastically and exhibited decreased colony pigmentation. Extensive conidiophores development was observed under salt stress; conidiophores rarely developed in the absence of salt stress. Under salt stress, yellow cleistothecium formation was inhibited, while glycerol and compatible sugars continued to accumulate. Among differentially expressed unigenes (DEGs), 733 of them were up-regulated while 1,619 unigenes were down-regulated. We discovered that genes involved in the accumulation of glycerol, the storage of compatible sugars, organic acids, pigment production, and asexual sporulation were differentially regulated under salt stress. These results provide further understanding of the molecular basis of osmoadaptive mechanisms of halophilic endophytic fungi.

## Introduction

Extremophilic microorganisms not only survive, but can grow optimally under rough conditions which are considered harsh and inhospitable for other life forms as well (Mesbah and Wiegel, 2012). Hypersaline environment is one of the examples of such extreme environments (Wood, 2015). Generally, high salinity represents high-osmotic stress, which triggers cytoplasm shrinkage and causes lethal damage to salt-sensitive microbes (Koch, 1984; Morris et al., 1986). However, some microbes learn to cope with these high salt concentrations by developing special strategies (Oren, 2002). In hypersaline environments, bacteria are considered to be the only populated microorganisms (Gunde-Cimerman et al., 2009); however, the report by Gunde-Cimermana et al. (2000) show the presence of fungi in saline environments. Since then, many fungal spp. were reported from different saline environments (Plemenitaš et al., 2008; Gunde-Cimerman et al., 2009).

Fungi use different strategies to overcome salt stress, such as morphological change (Zajc et al., 2013), reinforcement of cell walls, accumulation of osmolytes like glycerol, and change in genetic structures (Duran et al., 2010; Kralj Kuncic et al., 2010; Kis-Papo et al., 2014). Changes to salt stress at the transcriptomic level are still intricate, and the molecular mechanisms are not well-known. With the development of RNA-sequencing, it is easy to detect transcriptomic responses in a given species to varying experimental conditions (Schweder and Hecker, 2004; Dalmolin et al., 2012; Taymaz-Nikerel et al., 2016).

Next-generation sequencing technology (Illumina RNA-seq) enables highly sensitive and accurate quantification of expression, thus providing a myriad of transcript data with high resolution, high quality, and low costs (Riccombeni and Butler, 2012). It has been widely applied in non-model and model species, such as, *Aspergillus nidulans, Hortaea werneckii*, and *Wallemia ichthyophaga*, examining hundreds of stress-tolerance genes (Redkar et al., 1996; Petrovic et al., 2002; Zajc et al., 2013). Although, studies are carried out to understand salt stress-tolerance genes in fungi, there are only a few reports on response of endophytic fungi to high-salt stress. In this study, we made an attempt to isolate an endophytic fungus from a hypersaline region and evaluate its morphological and transcriptomic response under salt stress.

## Materials and methods

### Isolation and identification of endophytic fungal strain ZYD4

*Medicago sativa* L. grew in hypersaline environment (Huama lake region, Northern Shaanxi, China) was chosen as a source plant for the isolation of endophytic fungi. The endophytic strain ZYD4, was isolated using yeast extract-peptone-dextrose (YPD) agar (Difco) from the stems of *Medicago sativa* L. which were surface sterilized by following the protocols of Li et al. (2009) and Salam et al. (2017). For the identification, genomic DNA from strain ZYD4 was extracted by following the protocol of Hinrikson et al. (2005). ITS fragments of strain ZYD4 were amplified using the primers, ITS1 and ITS4 (White et al., 1990).

The analysis of sequences was done at Blast-n site at NCBI server (http://www.ncbi.nlm.nih.gov/BLAST).

The phylogenetic tree was constructed by neighbor-joining (NJ) (Saitou and Nei, 1987) method using MEGA 5.0 software package (Tamura et al., 2011) after multiple alignment of the sequences using CLUSTAL_X program (Thompson et al., 1997). Kimura's two parameter model was used to calculate evolutionary distance matrices (Kimura, 1980). Bootstrap analysis was performed with 1,000 replications (Felsenstein, 1985).

### Morphological response of endophytic strain ZYD4 to salt stress

The morphological response of endophytic strain ZYD4 to salt stress was evaluated at salt concentrations ranging from 0 to 4.5 M (at an interval of 1.5 M) using YPD agar at 28°C for 6 days.

### Evaluation of salt stress on the biosynthesis of pigment and accumulation of glycerol and compatible sugars

To evaluate salt stress on the biosynthesis of pigment and accumulation of glycerol and compatible sugars, we grew strain ZYD4 in the presence and absence of 3 M NaCl. The collected mycelium was washed three times with sterilized distilled water and freeze-dried at −30°C.

The mycelium was thoroughly ground in liquid nitrogen. The pigment was extracted with methanol (Liu et al., 2015) and monitored by UV–visible spectrophotometer (Shimadzu UV-1750, Japan) using β-Carotene and flavoglaucin as standard.

Glycerol and compatible sugars were extracted in sterilized distilled water using sonicator (350 W, 1 min) performed on the ice bath followed by centrifugation at 10,000 rpm for 10 min at 4°C. All extracts were filtered through a 0.22-μm filter before the examination. Glycerol and compatible sugars were determined using UV–visible spectrophotometer (Shimadzu UV-1750, Japan) by following the protocols of Kuhn et al. (2015) and Laurentin and Edwards (2003).

### RNA extraction, library construction, and illumina sequencing

We statically grew endophytic strain ZYD4 on YPD broth at 28°C for 6 days. The fungal mycelium was harvested and induced under salt stress by adding 3 M NaCl at 28°C for 30 min. Total RNA from salt-stress induced and non-induced mycelium was extracted using the Trizol reagent (Invitrogen, Carlsbad, CA, USA), according to manufacturer's instructions. The RNA samples were treated with DNase I for 30 min at 37°C to remove genomic DNA contamination. The quantity and integrity of the total RNAs were verified using an Agilent 2100 bioanalyzer. The cDNA libraries were developed according to manufacturer's instructions (Illumina, Inc., San Diego, CA, USA), and sequenced on the Illumina HiSeq 2000 platform at Beijing Genomics Institute (Shenzhen, China).

### *De novo* assembly and analysis

Before the assembly, raw reads were cleaned by removing adaptor sequences, low-quality reads, and reads with unknown nucleotides >5%. All clean reads were *de novo* assembled into contigs using the short-read assembling program Trinity (Grabherr et al., 2011). The contigs were pooled to build into de Brujin graphs by Chrysalis. Contigs from the same transcript and the distances between these contigs were detected by a paired-end sequencing strategy. Unigenes from each samples assembly were further processed with sequence-clustering software to remove sequence splicing and redundancy. The unigenes were divided into clusters (CL prefix) and singletons (unigene prefix) by gene family clustering.

### Functional annotation and differential expression analysis

All unigenes were compared against NCBI non-redundant protein database (NR), the NCBI non-redundant nucleic acid database (NT), the Swiss-Prot database, the Clusters of Orthologous Groups (COG) database, and the Kyoto Encyclopedia of Genes and Genomes (KEGG) database with an *E* < 10^−5^. The best aligned results were applied to determine sequence direction of unigenes. The Gene Ontology (GO) was analyzed with Blast2GO software (Conesa et al., 2005). The GO functional classification was performed using WEGO software (Ye et al., 2006). Protein coding region prediction of the unigenes was searched by BLASTx (*E* < 0.00001) against NCBI NR, Swiss-Prot, KEGG, and COG. When unigene was not aligned to any of the above databases, ESTS was used to predict its coding regions and ascertain its sequence direction (Iseli et al., 1999). Differentially expressed unigenes were filtered using a threshold of false discovery rate (FDR ≤ 0.001) and an absolute log2 ratio ≥1. These differential expression unigenes were further mapped onto known pathways using the KEGG pathway annotation.

### qPCR

qPCR was performed to validate the differential expression of randomly selected genes involved in salt-tolerance on a 7500 Real-Time PCR System (Applied Biosystems, USA) under the following conditions: 95°C for 30 s, followed by 40 cycles of 95°C for 15 s and 60°C for 40 s. The q-PCR mixture (20 μl) comprised of 10 μl of SYBR® Select Master Mix (Applied Biosystems, USA), 10 ng of cDNA, primers (0.4 μM each; Table S1), and RNase-free water. All PCR reactions were run in duplicate for each gene along with the endogenous 18S rRNA reference gene. The 2^−ΔΔCt^ method (Livak and Schmittgen, 2001) was employed to calculate the gene expression levels in salt with and without treated samples.

## Results

### Identification of endophytic fungal strain ZYD4

**Figure 1:**
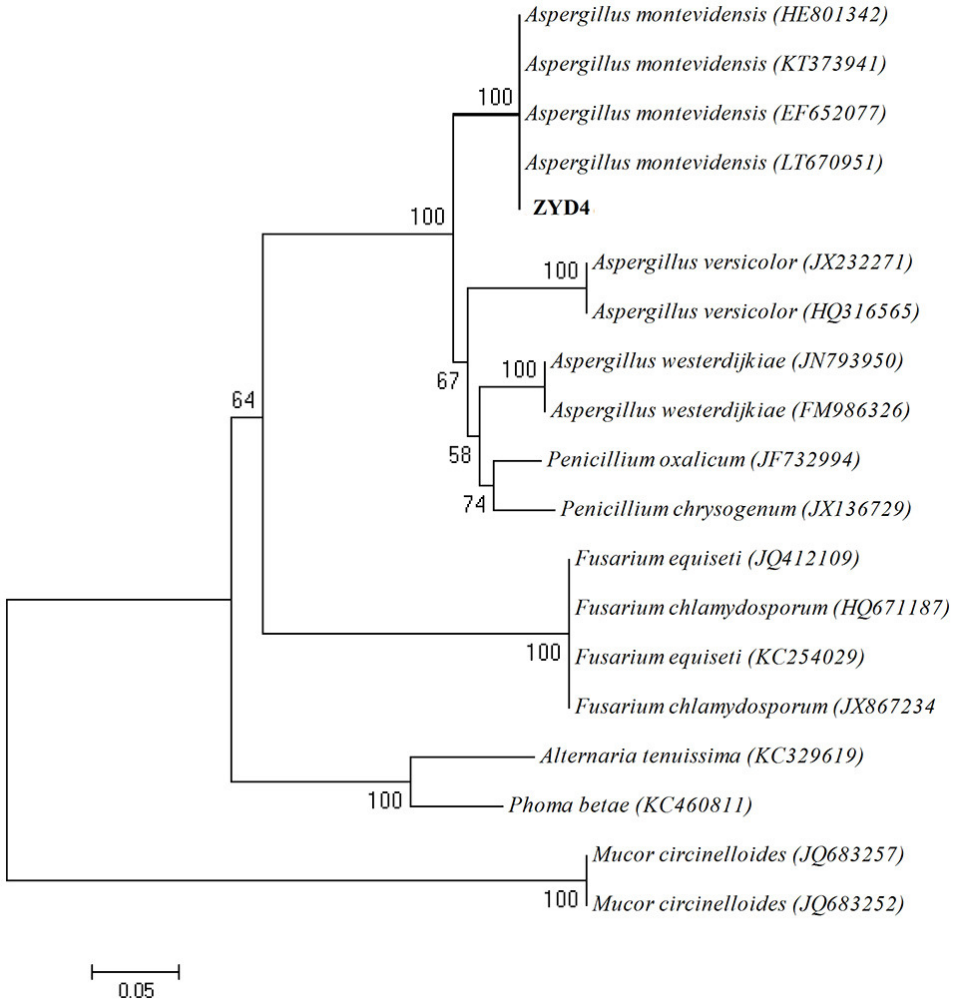
Neighbor-joining tree showing the phylogenetic relationship of the strain ZYD4. Boot strap values (expressed as percentages of 1,000 replications) >50% was given at the node.

The BLAST result showed that, endophytic strain ZYD4 shared 100% similarity with *Aspergillus montevidensis*. The obtained sequences were submitted to GenBank under the accession number MF062488. In NJ tree (Figure 1) strain ZYD4 was grouped with *A*. *montevidensis*. Based on the above, strain ZYD4 was identified as *A*. *montevidensis*. The strain ZYD4 was deposited in China General Microbiological Culture Collection Center under the deposition number CGMCC 3.15762.

### Morphological response of *A. montevidensis* ZYD4 under salt stress

**Figure 2:**
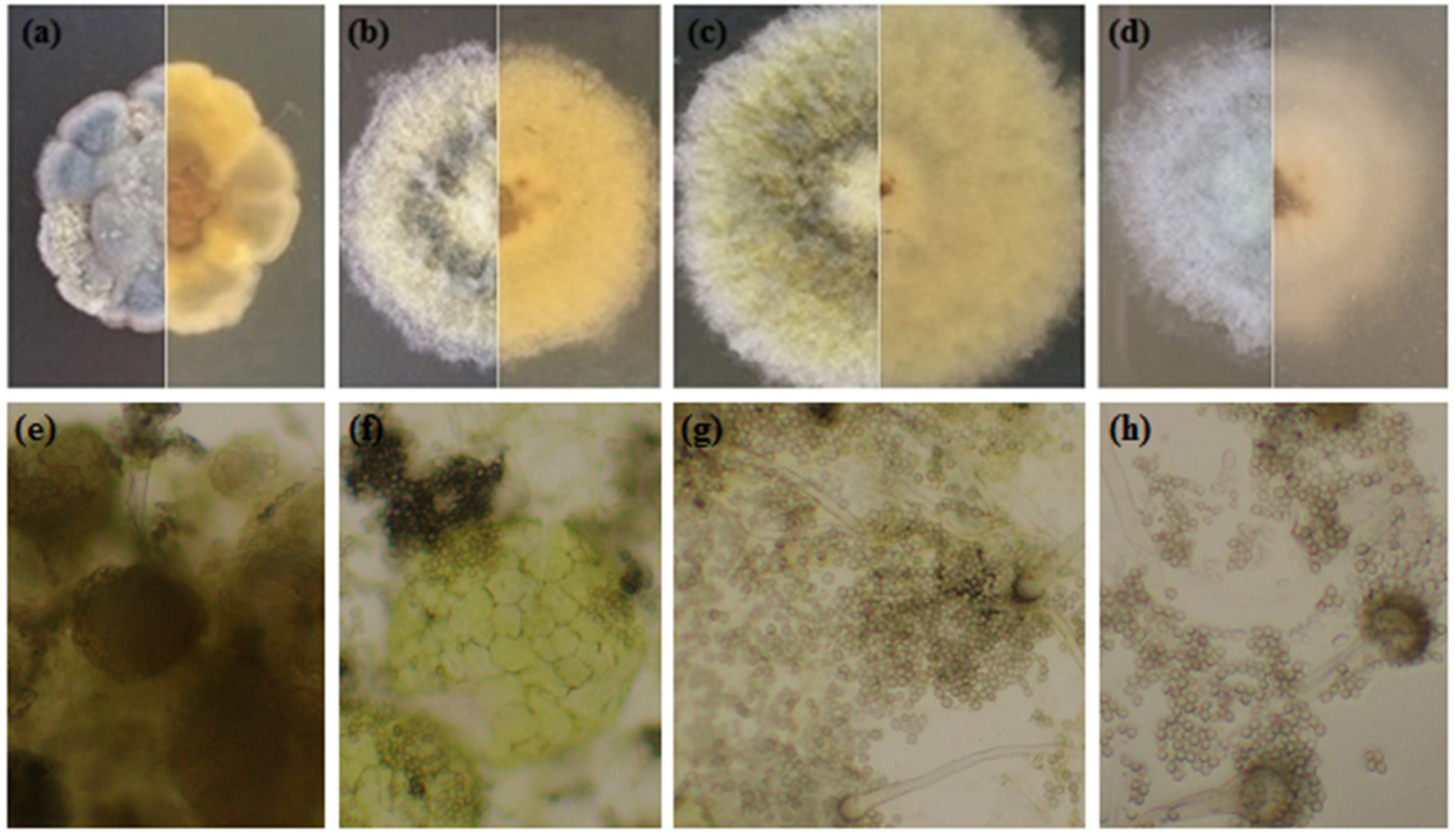
Morphological profile of *A*. *montevidensis* ZYD4 on solid medium supplemented with varying concentrations of NaCl: **(a,e)** without NaCl; **(b,f)** 1.5 M NaCl; **(c,g)** 3 M NaCl; **(d,h)** 4.5 M NaCl.

Under salt stress, the morphological characters such as colony shape, color, size, and conidial head formation of *A. montevidensis* ZYD4 changed (Figures 2b–d, Table 1). Under salt stress, the mycelium was loose while compact in absence of salt stress. The width of hyphae decreased under salt stress (Table 1). Under salt stress, *A*. *montevidensis* ZYD4 colonies were olive green in the center and white at the margin while in the absence of salt stress, colonies were yellow to gray black in center and golden yellow at the margin. The conidial head was extensively developed in the presence of salt stress, while rarely formed in the absence of salt stress. Formation of yellow cleistothecium was dramatically inhibited with an increase of salt stress (Figures 2e–h, Table 1).

**Table 1:** Morphological profile of *A*. *montevidensis* ZYD4 on solid medium supplemented with varying salt concentration.

**Isolate**	**NaCl in media (M)**	**Morphology**
*A. montevidensis* ZYD4	0	Compact mycelium; colony was yellow to gray black in the center and golden yellow at the margin; the branched hyphae are septate, and measure about 2.5–6.25 μm in width; conidiophores chain rarely formed from swelling conidial heads; spherical conidia of 3.75 × 3.75 μm size; yellow cleistothecia extensively developed; ascospores measure about 1.67–2.77 × 2.08–3.15 μm.
	1.5	Loose mycelium; colony was yellow to gray black in the center and gray-black at the margin; the branched hyphae are septate, and measure about 2.5–6.25 μm in width; gray pigmented conidial heads developed; spherical/oval conidia of 2.5–4.0 × 3.75–5.0 μm size; yellow cleistothecia extensively produced; ascospores measure about 1.67–2.77 × 2.08–3.15 μm.
	3	Loose mycelium; colony was olive green in the center and white at the margin; the branched hyphae are septate and measure about 2.5–6.25 μm in width; olive green/white pigmented conidial heads extensively developed; spherical/oval conidia of 2.5–4.0 × 3.75–5.0 μm size.
	4.5	Loose and white mycelium; the branched hyphae are septate and measure about 2.0–2.5 μm in width; gray-green pigmented conidial heads extensively developed; spherical/oval conidia of 2.5–4.0 × 3.0–5.0 μm size.

### Evaluation of salt stress on the biosynthesis of pigment and accumulation of glycerol and compatible sugars

**Figure 3:**
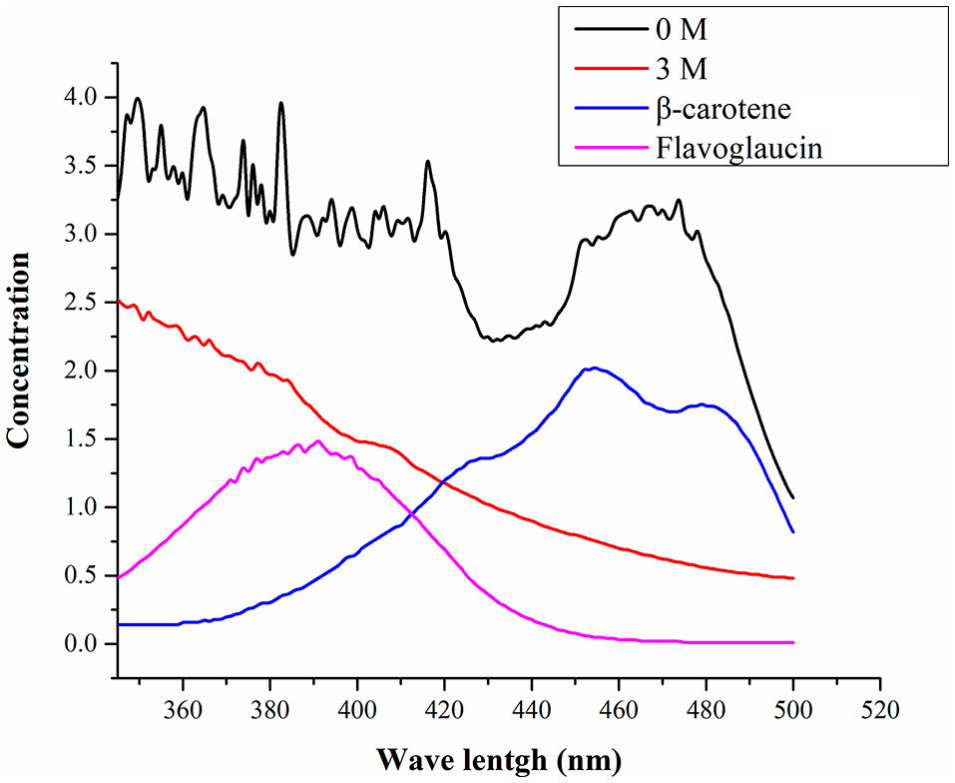
UV-visible spectra of the pigments.

Figure 3 shows UV-visible spectra of pigment biosynthesis analysis which suggest that, *A. montevidensis* ZYD4 grown under salt stress did not show any pigment production, while in absence of salt stress pigment production was observed.

**Figure 4:**
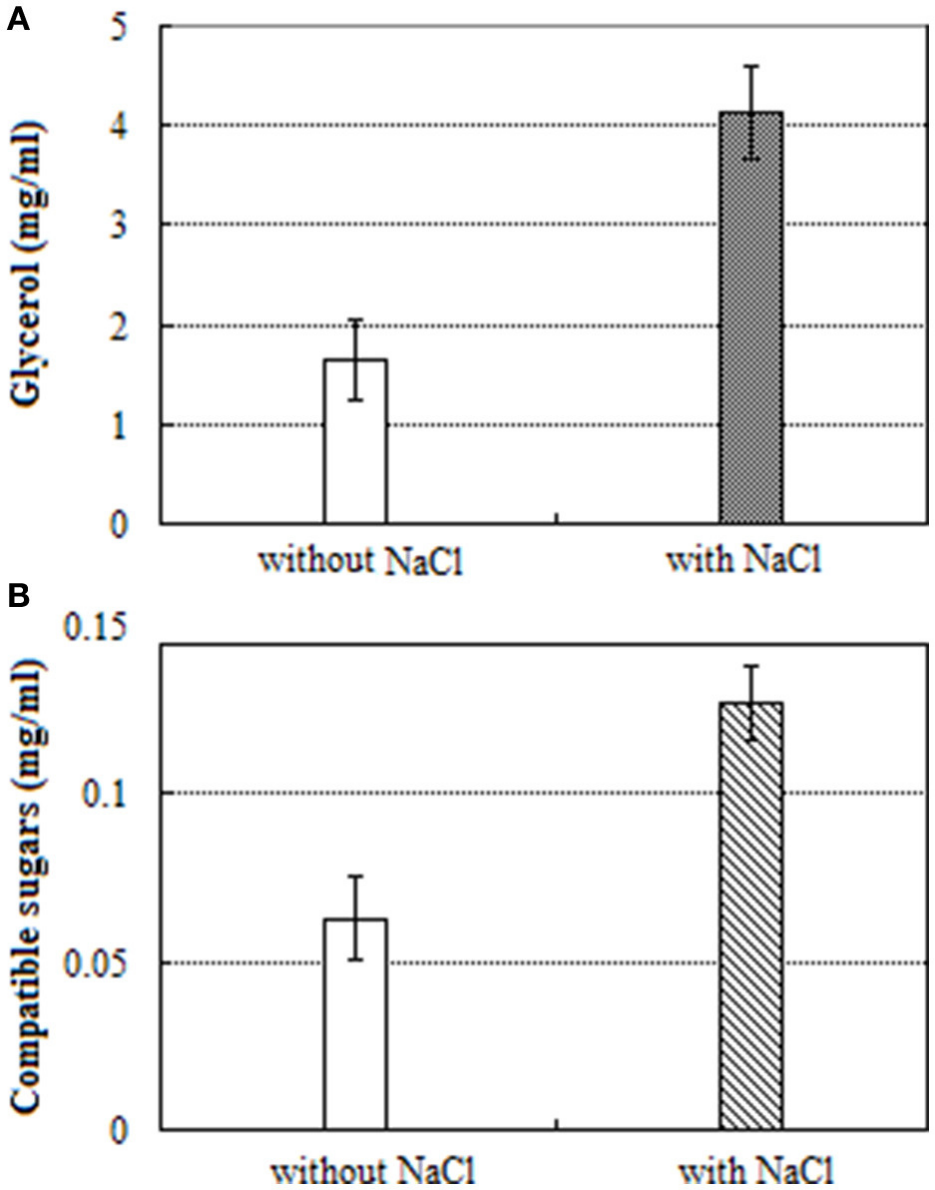
UV-visible spectra of: **(A)** glycerol and **(B)** compatible sugars.

The content of glycerol in salt treated mycelium was 2.5-fold high when compared to salt untreated mycelium (Figure 4A). Similarly, the contents of total compatible sugars in salt treated mycelium was 2.0-fold high when compared to salt untreated mycelium (Figure 4B).

### Illumina sequencing and *de novo* assembly

To explore osmoadaptive strategies, cDNA libraries of *A*. *montevidensis* ZYD4 (from salt with and without treated mycelium) were sequenced using the Illumina Hiseq 2000 platform. The raw reads obtained from salt with and without induced mycelium were submitted into GenBank under the accession numbers SRX1794941 and SRX1794940 respectively. After the removal of the adapter sequences, unknown, and low-quality sequences about 104,004,358 clean reads (average length 90 nt) were obtained from raw reads with GC percentage between 50.57 and 51.63%.

These clean reads were *de novo* assembled (Table 2) resulting in 34,341, and 28,503 contigs for salt without and with treated samples, with a mean length of 595, and 657 nt and an N50 length of 1,717, and 1,789 nt respectively.

**Table 2:** Summary of *A. montevidensis* ZYD4 transcriptome assembly.

	***A. montevidensis* with NaCl**	***A. montevidensis* without NaCl**
Number of contings	28,503	34,341
Total length of contings (nt)	18,739,029	20,437,448
Mean length conting (nt)	657	595
N50 contig length (nt)	1789	1717
Total unigenes	24,157	27,797
Total length of unigenes (nt)	30,268,028	33,330,542
Mean length of unigene (nt)	1253	1199
N50 unigene length (nt)	2268	2283
Distinct clusters	8,643	9,765
Distinct singletons	15,514	18,032

After further clustering and assembly, 27,797 unigenes for salt untreated sample and 24,157 unigenes for salt treated sample, with an average length of 1,199, and 1,253 nt, and N50 length of 2,283, and 2,268 nt respectively were obtained. There were 46,464 unigenes with the length ≥500 nt, and 41,844 unigenes with the length ≥1,000 nt. The length distribution of these contigs and unigenes were shown in Table 2.

### Functional annotation

**Figure 5:**
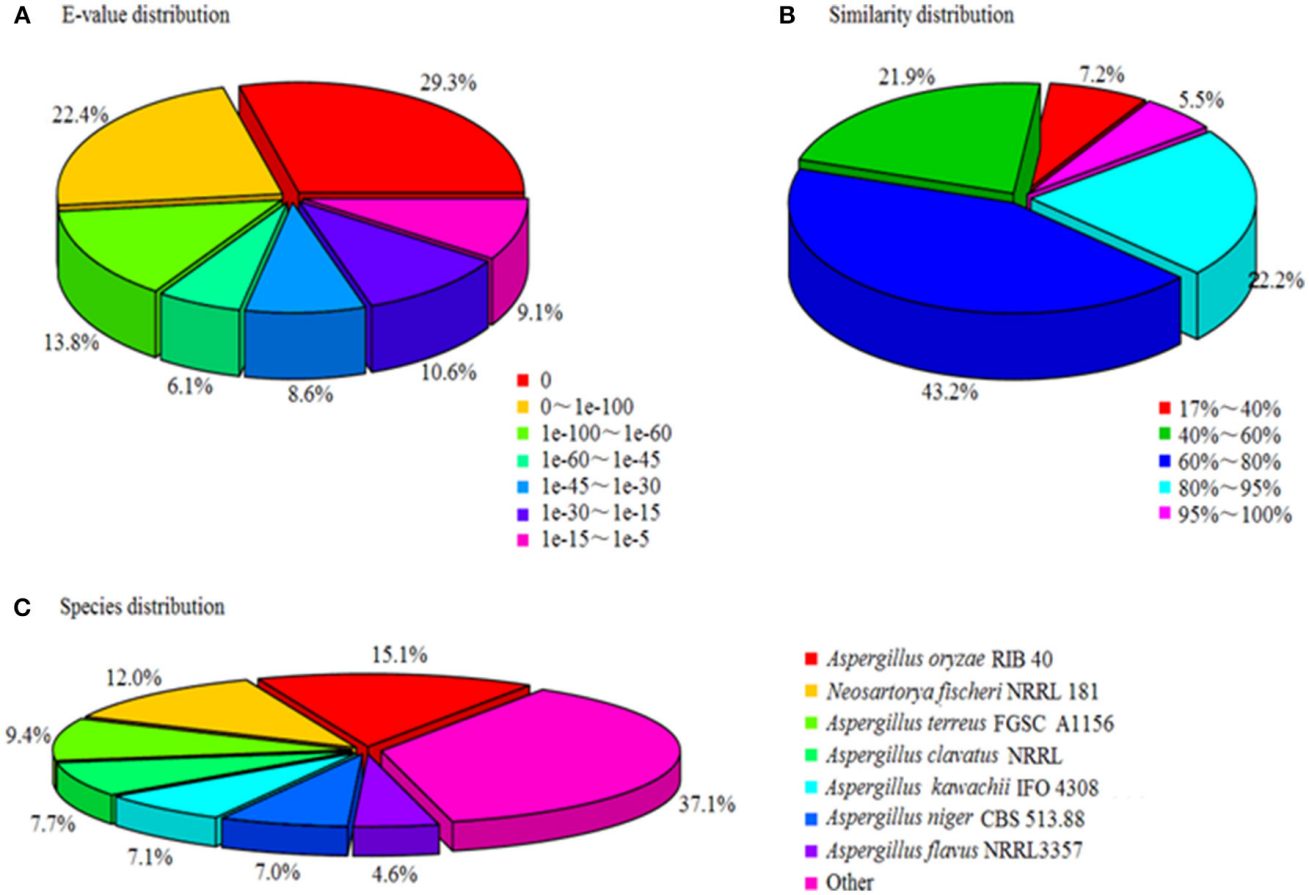
Characteristics of homology search of unigenes against Nr databases. **(A)** *E*-value distribution of BLASTx matches to the unigenes against Nr; **(B)** Similarity in distribution of BLASTx matches to the unigenes against Nr; **(C)** Species distribution of BLASTx matches to the unigenes against Nr.

A total of 18,267 unigenes were annotated, among them 17,713, 12,197, 12,018, and 9,029 showed high similarities to the known genes in NR, SwissProt, KEGG, and COG databases. The NR annotation and E-value distribution (Figure 5A) of significant hits (*E* < 1.0 × 10^−45^) showed that 71.6% of the sequences had strong homologies, 19.2% of the sequences had a high degree of homologies with *E* < 1.0 × 10^−15^, and 70.9% of the sequences had high similarity >60%, while 21.9% showed a similarity range between 40 and 60% (Figure 5B). Nearly 62.9% of the annotated unigenes assigned to top BLAST hits were closely related to *Aspergillus* species, namely *Aspergillus oryzae* (15.1%), *Aspergillus terreus* (9.4%), *Aspergillus clavatus* (7.7%), *Aspergillus kawachii* (7.1%), *Aspergillus niger* (7%), and *Aspergillus flavus* (4.6%; Figure 5C).

### GO and COG classification

**Figure 6:**
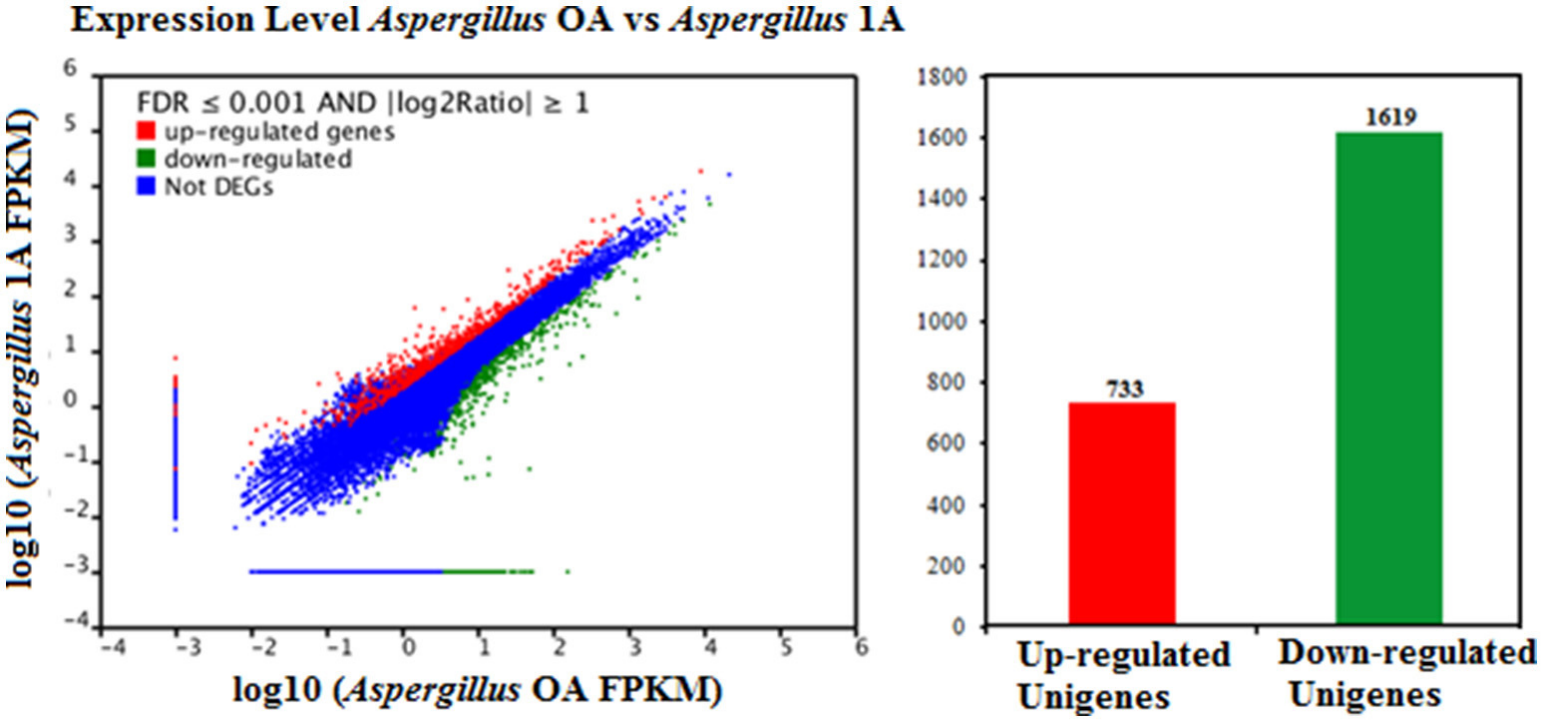
Comparison of gene expression levels in the control and the salt-treated samples. *Aspergillus* OA represents salt untreated sample, and *Aspergillus* 1A represents salt treated samples. Fragments Per Kilobase of transcript per Million fragments mapped abbreviated as FPKM.

**Figure 7:**
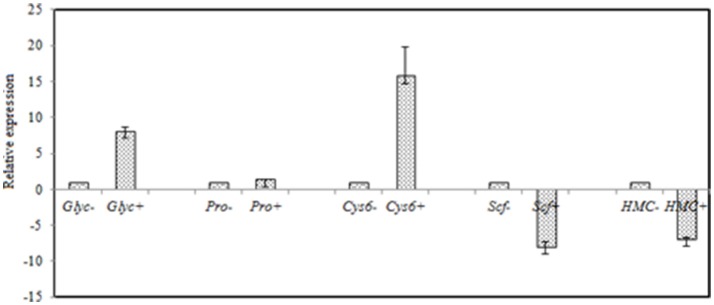
RT-qPCR validation of selected genes with significant differential expression in the control and the salt-treated samples. RT-qPCR data are mean ± *SD* from three biological replicates. “−,” and “+” indicates *A. montevidensis* was induced without, and with 3 M NaCl. DEGs putatively encoded glycerol-3-phosphate dehydrogenase (*Glyc*), proline oxidase (*Pro*), Zn(II)2Cys6 transcription factor (*Cys6*), SCF ubiquitin ligase (*Scf*), and hydroxymethylglutaryl-CoA (HMG-CoA) synthase (*HMC*).

**Figure 8:**
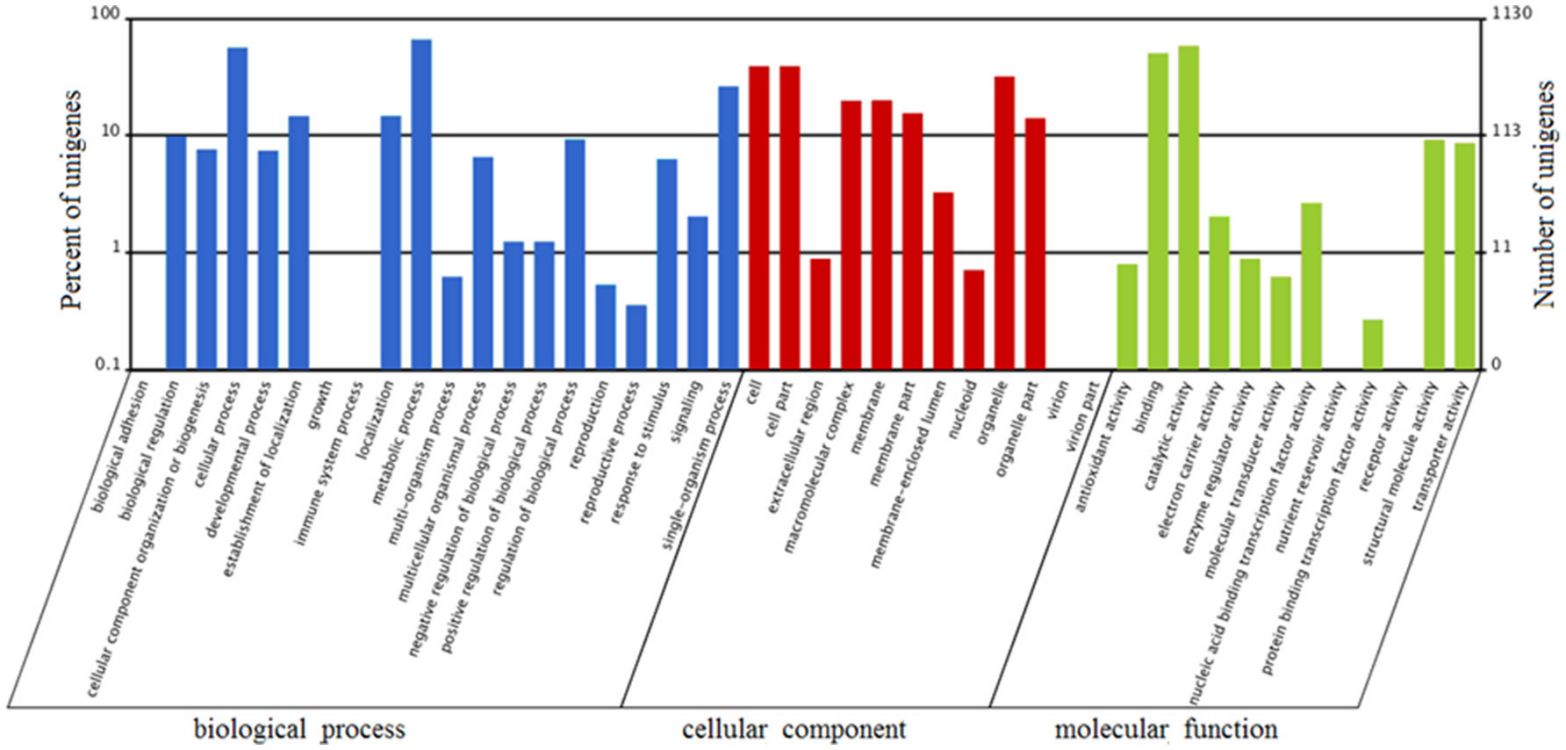
Gene Ontology (GO) terms enrichment of unigenes of *A. montevidensis* ZYD4 at transcriptome level. The results were summarized in three main categories: biological process, cellular component, and molecular function. The right y-axis presents the number of unigenes in the category, while the left y-axis presents the percentage of a specific category of unigenes in that category.

**Figure 9:**
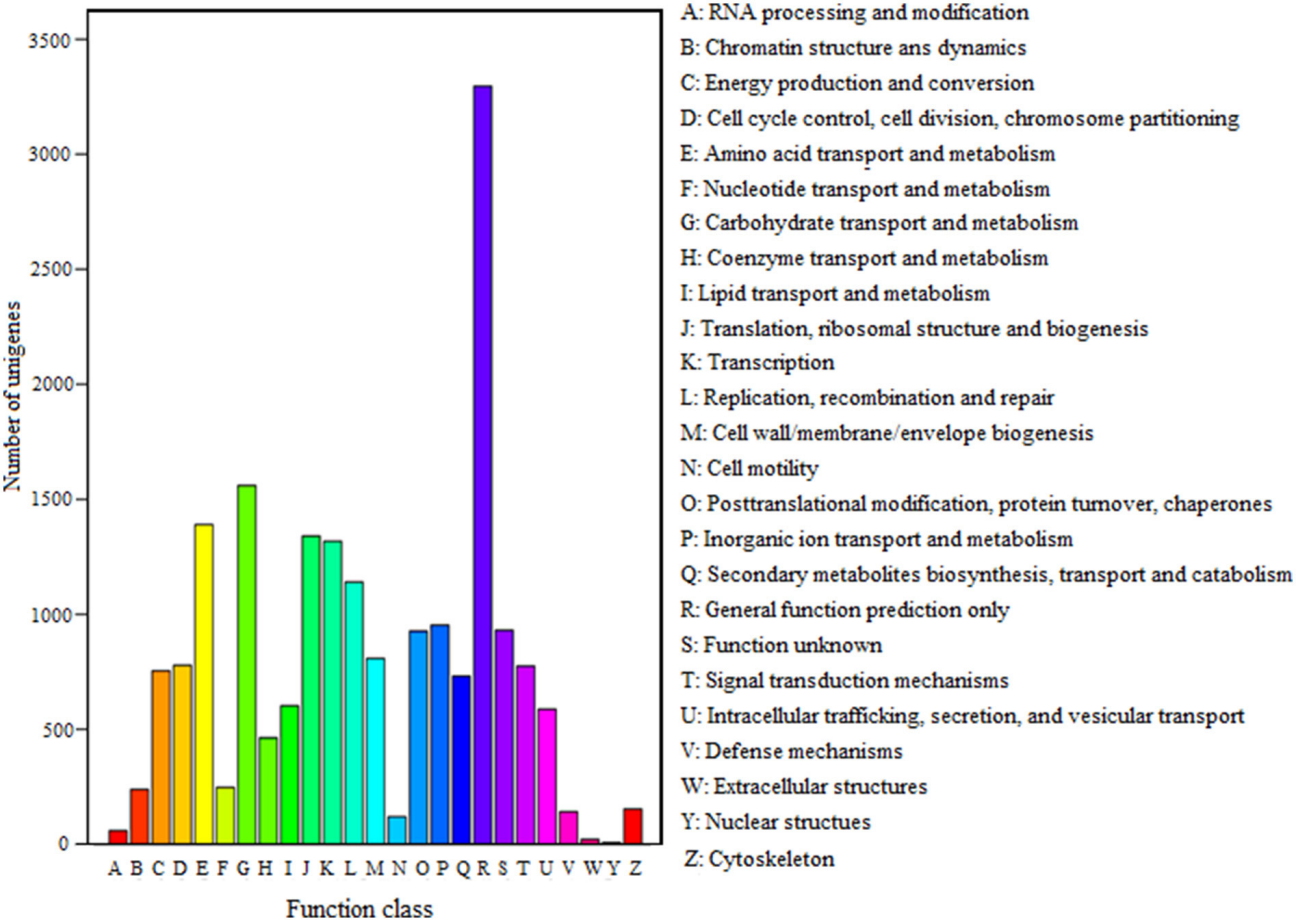
COG classification of unigenes. A total of 7,576 unigenes had a COG classification among the 25 categories. The capital letters on the x-axis indicate the COG categories as listed on the right of the histogram.

FDR ≦ 0.001 and log2 ratio ≧ 1 as the threshold were used to verify the significance of gene expression differences. Based on these criteria, a total of 2,352 unigenes were differentially expressed in presence and absence of salt stress. Among differentially expressed unigenes (DEGs), 733 unigenes were up-regulated while 1,619 unigenes were down-regulated (Figure 6). The expression patterns of some DEGs were validated by RT-PCR (Figure 7). DEGs were assigned to 44 GO terms consisting of three domains namely biological process, molecular function, and cellular component (Figure 8). The representative distributions of the GO terms for biological processes include cellular process, metabolic process, and single-cell process. A majority of molecular function was composed of cells, cell part, and organelle. Cellular component mainly included catalytic activity, and binding. To verify the effectiveness of annotation, COG classification of DEGs was performed resulting in 25 COG groups. Of these, general function prediction cluster (16.7%) was the dominant group, followed by replication, recombination and repair (8.66%), translation, ribosomal structure and biogenesis (7.45%), and RNA processing and modification (0.64%; Figure 9).

### KEGG pathway analysis

To analyze the salt stress functions of DEGs, we mapped DEGs to 100 reference canonical pathways in KEGG database. The significantly enriched DEGs were mainly involved in 20 pathways, such as metabolic pathways (ko01100, 409 unigenes, 32.8%), biosynthesis of secondary metabolites (ko01110, 202 unigenes, 16.2%), ribosome (ko03010, 117 unigenes, 9.38%), starch and sucrose metabolism (ko00500, 79 unigenes, 6.34%), amino sugar and nucleotide sugar metabolism (ko00520, 68 unigenes, 5.45%), oxidative phosphorylation (ko00190, 56 unigenes, 4.49%), glycolysis/gluconeogenesis (ko00010, 29 unigenes, 2.33%), glycerophospholipid metabolism (ko00564, 26 unigenes, 2.09%), and cell cycle (ko04111 and ko04113, 38 unigenes, 1.6%). The result of KEGG pathway analysis showed multiple significant enriched pathways which were implicated in response to high salt stress in *A*. *montevidensis* ZYD4.

### Genes involved in the development of asexual sporulation

After exposure to high-salt stress, *A*. *montevidensis* ZYD4 extensively developed conidiophores and asexual spores (Figure 2). Correspondingly, unigenes homologous to known genes, controlling asexual development of *A. montevidensis* were also up-regulated (Table 3).

**Table 3:** Summary of some differently expressed genes of *A*. *montevidensis* in response to high-salt stress.

**Unigene ID**	**Putative function**	**Log2 fold**	**Pathway**
**GLYCEROL**			
CL3405	glycerol-3-phosphate dehydrogenase	+1.4	Glycolysis
Unigene 3991	glycerol dehydrogenase	-1.4	Glycolysis
Unigene 788	glycerol dehydrogenase (NADP^+^)	+1.1	Glycerolipid metabolism
Unigene 2376	aldehyde dehydrogenase (NAD^+^)	+1.1	Glycerolipid metabolism
Unigene 9950	diacylglycerol O-acyltransferase	-11.0	Glycerolipid metabolism
CL1785	triacylglycerol lipase	-2.0	Glycerolipid metabolism
Unigene 1646	lysophospholipase	+1.1	Glycero-phospholipid metabolism
Unigene 4940	phospholipase C	+1.3	Glycero-phospholipid metabolism
CL944	glycerol-3-phosphate dehydrogenase (NAD^+^)	+1.4	Glycero-phospholipid metabolism
CL3405	glycerol-3-phosphate dehydrogenase	+1.4	Glycero-phospholipid metabolism
Unigene3569	choline kinase	+1.2	Glycero-phospholipid metabolism
Unigene7877	phospholipase D	-1.4	Glycero-phospholipid metabolism
**COMPATIBLE SUGARS**			
CL184	6-phosphofructo-2-kinase	+2.2	Glycolysis
CL1930	trehalose-phosphate synthase	+1.0	Starch and sucrose metabolism
CL1942	mannose-6-phosphate isomerase	+1.5	Starch and sucrose metabolism
CL1896	hexokinase	+1.4	Glycolysis
Unigene 9250	glucose-6-phosphate isomerase	-11.2	Glycolysis
CL1568	fructose-bisphosphate aldolase	-13.1	Glycolysis
Unigene 8898	triose-phosphate isomerase	-12.5	Glycolysis
CL1944	glyceraldehyde 3-phosphate dehydrogenase	-13.9	Glycolysis
Unigene 8982	Phosphoglycerate kinase	-11.9	Glycolysis
Unigene 8902	enolase	-13.1	Glycolysis
Unigene 8995	pyruvate kinase	-11.8	Glycolysis
Unigene 8882	phosphoenolpyruvate carboxykinase	-11.5	TCA cycle
Unigene 8970	pyruvate dehydrogenase	-12.2	TCA cycle
Unigene 8600	dihydrolipoamide dehydrogenase	-12.2	TCA cycle
Unigene 8566	citrate synthase	-11.5	TCA cycle
Unigene 8945	ATP-citrate synthase	-12.5	TCA cycle
Unigene 9020	aconitate hydratase	-12.1	TCA cycle
Unigene 9386	isocitrate dehydrogenase	-11.4	TCA cycle
Unigene 8508	2-oxoglutarate dehydrogenase E1	-11.9	TCA cycle
Unigene 9002	2-oxoglutarate dehydrogenase E2	-11.8	TCA cycle
Unigene 8600	dihydrolipoamide dehydrogenase	-12.2	TCA cycle
Unigene 10103	succinate dehydrogenase (ubiquinone) iron-sulfur subunit	-10.8	TCA cycle
Unigene8987	malate dehydrogenase	-11.7	TCA cycle
**ORGANIC ACIDS**			
Unigene 3431	proline oxidase	+3.6	Proline cycle
Unigene 1427	pyrroline-5-carboxylate dehydrogenase	+1.9	Proline cycle
CL601	glutamate decarboxylase	+5.0	Proline cycle
Unigene 8818	glutamate dehydrogenase	-13.8	Proline cycle
Unigene 9009	glutamine synthetase	-11.6	Proline cycle
**YELLOW PIGMENT**			
Unigene 4069	geranylgeranyl diphosphate synthase	-1.1	HMG-CoA pathway
Unigene 9164	RAB proteins geranylgerany ltransferase component A	-11.5	HMG-CoA pathway
Unigene 9310	farnesyl pyrophosphate synthetase	-11.4	HMG-CoA pathway
Unigene 8875	hydroxymethylglutaryl-CoA (HMG-CoA) synthase	-13.1	HMG-CoA pathway
**ASEXUAL SPORULATION**			
Unigene 11005	Zn(II)2Cys6 transcription factor	+4.4	Cell cycle
CL66	G1/S-specific cyclin Cln1	+1.5	Cell cycle
CL2000	cell cycle arrest protein BUB2	+1.3	Cell cycle
CL2756	Guanine nucleotide exchange factor LTE1	+1.1	Cell cycle
CL707	DNA replication licensing factor mcm7	+1.0	Cell cycle
Unigene 5683	Spindle assembly checkpoint component MAD1	+1.6	Cell cycle
Unigene 9493	SCF ubiquitin ligase	-11.1	Cell cycle
Unigene 9618	DNA replication licensing factor Mcm5	-3.0	Cell cycle
Unigene 7664	nuclear condensin complex subunit Smc4	-2.9	Cell cycle
CL1202	Spore-wall fungal hydrophobin dewA	+1.6	Cell cycle

### Genes involved in the biosynthesis of yellow pigment

The yellow pigment produced by *A*. *montevidensis* ZYD4 was decreased under salt stress. DEGs related to the biosynthesis of carotenoids in *A*. *montevidensis* ZYD4 was summarized in Table 3. It was observed that, unigenes homologous to geranylgeranyl diphosphate synthase, hydroxymethylglutaryl-CoA (HMG-CoA) synthase, and farnesyl pyrophosphate synthase were down-regulated by 1.1-, 13.1-, and 11.4-fold respectively.

### Genes involved in the accumulation of glycerol

Under salt stress, the expression of one unigene encoding glycerol-3-phosphate dehydrogenase (which participates in the glycerol biosynthetic pathway) was elevated by 1.4-fold (Table 3), however, one unigene encoding glycerol dehydrogenase (which converts glycerol into dihydroxyacetone) showed 1.4-fold decrease of expression. Unigenes coding for enzymes responsible for glycerol biosynthesis were up-regulated by 1.1- to 1.4-fold, while unigenes encoding diacylglycerol O-acyltransferase, triacylglycerol lipase, and phospholipase D (these participate in the further transformation of glycerol) were down-regulated by 1.4- to 11.0-fold. These results indicated that glycerol was accumulated in *A*. *montevidensis* ZYD4 to create a cellular osmotic equilibrium in high-salt conditions.

### Genes involved in the biosynthesis of compatible sugars

Under salt stress, expression of unigenes homologous to hexokinase, 6-phosphofructo-2-kinase, trehalose-phosphate synthase, and mannose-6-phosphate isomerase increased by 1.0- to 2.2-fold (Table 3). Remarkably, 19 DEGs for the further conversion of glucose-6-phosphate into other intermediates via the down-stream pathway of glycolysis and TCA cycle were suppressed up to 10.8- to 13.9-fold. In addition, unigenes responsible for the further transformation of acetyl-CoA (a metabolic intermediate of sugar) into fatty acids, and carotenoids were down-regulated. The above results obtained were consistent with UV-visible spectroscopic analysis (Figure 4B).

### Genes involved in the biosynthesis of organic acids

Unigenes of proline oxidase and pyrroline-5-carboxylate dehydrogenase increased the expression by 3.6, and 1.9-fold respectively in salt induced samples (Table 3). However, unigenes of glutamate dehydrogenase, and glutamine synthetase (can divide glutamate into glutamine, ${\text{NH}}_{4}^{+}$ and 2-oxo-glutarate) was suppressed more than 11-fold. Moreover, expression level of one unigene, encoding glutamate decarboxylase, was augmented by 5-fold. A detailed up and down regulated genes involved in biosynthesis of organic acids were mentioned in Table 3.

## Discussion

Survival and growth of microorganisms in saline environments require numerous morphological ecotypes and adaptations. Salt stress causes changes in colony morphology, colony pigmentation, and cell wall structure (Kralj Kuncic et al., 2010). Hence, in the present study we evaluated the morphological changes caused by salt stress. In the absence of salt stress, mycelium of *A*. *montevidensis* ZYD4 was compact while loose in the presence of salt stress. Further, the hyphae width was reduced under salt stress. Similar to our results, fungal hyphae growth was effected under salt stress (Matsuda et al., 2006). Under salt stress, *A*. *montevidensis* ZYD4 conidial heads were extensively developed while without salt stress conidial heads were rarely formed.

It is widely known that carotenoids and flavoglaucin are responsible for yellow, bright red, and orange hues of fungal colonies (Davoli and Weber, 2002; Christaki et al., 2013; Dufosse et al., 2014). *A*. *montevidensis* ZYD4 subjected to salt stress showed drastic change in colony color. Under salt stress, colonies of *A*. *montevidensis* ZYD4 were olive green in the center, and white at the margin. Colonies without salt stress, were yellow to gray black in the center, and golden yellow at the margin. Similar to our result, Kralj Kuncic et al. (2010) observed change in the colony color when *Wallemia* sp. subjected to salt stress.

The pigment production showed drastic variation under salt stress. The methanolic extract obtained from *A*. *montevidensis* ZYD4 grown in absence of 3 M NaCl showed peaks from 400 to 490 nm, indicating the presence of carotenoids (Klassen and Foght, 2008; Liu et al., 2015), while strain ZYD4 grown in presence of 3 M NaCl did not show any significant peak, indicating the absence of carotenoid. Similar to our results, Plemenitaš et al. (2008) found decreased pigment production in *H. werneckii* subjected to high salinity. Further, unigenes homologous to geranylgeranyl diphosphate synthase, hydroxymethylglutaryl-CoA (HMG-CoA) synthase, and farnesyl pyrophosphate synthase were down-regulated under salt stress.

Generally, enhanced expression of these genes increases fungal carotenoid content, which are made from acetyl-CoA through the HMG-CoA pathway (Davoli and Weber, 2002; Alcaino et al., 2014; Nagy et al., 2014). Therefore, down-regulated expression of these unigenes showed decreased synthesis of pigments in *A. montevidensis* ZYD4, and in turn regulated accumulation of intracellular glucose-6-phosphate by slowing down the metabolic flow of acetyl-CoA. The results suggest that the decrease of pigments was an osmoadaptive strategy.

Cleistothecium formation was dramatically inhibited with an increase of salt stress (Figures 2e–h, Table 1), suggesting that hypersaline condition triggered a transit from sexual to asexual state. The enhanced asexual development in *A*. *montevidensis* ZYD4 was critical in the life cycle being the primary means for survival under salt stress. Similar effect was observed in the marine-derived *Aspergillus glaucus* under high-salt stress (Liu et al., 2017).

In this study, we also evaluated genes involved in the development of asexual sporulation. The unigene 11005 encoding a Zn(II)_2_Cys_6_ transcription factor was upregulated by 4.4-fold. This factor plays a crucial role for asexual sporulation in different filamentous fungi (Vienken and Fischer, 2006; Chung et al., 2013; Gil-Duran et al., 2015; Son et al., 2016). Seven unigenes with significant identities to *CLN3, CIB3/4, CIB1/2, Bub2, Lte1, Mcm7*, and *Mad1* required for cell cycle progression through mitosis were up regulated by 1.0- to 2.3-fold, except for unigene 9493, encoding for ubiquitin ligase (E3) complex SCF subunit which was significantly down-regulated by 11.1-fold.

Unlike halophilic Archaea, halotolerant fungi do not accumulate high internal ion concentrations when grown in hypersaline conditions, but rather they counterbalance the osmotic imbalance by the accumulation of polyols (Kogej et al., 2005). Some studies suggest that glycerol accumulation is necessary for living cells to keep osmotic homeostasis and alleviate adverse effects of toxic Na^+^ ions (Kogej et al., 2007). In this study, we found that unigenes involved in the accumulation of glycerol were extensively enhanced in expression, however unigenes participated in the transformation of glycerol were down-regulated, suggesting that glycerol was accumulated in cells upon the hypersaline shock. Expression of two unigenes, encoding for glycerol-3-phosphate dehydrogenase, and 6-phosphofructo-2-kinase was augmented by 1.4, and 2.2-fold respectively. These unigenes are essential for glycerol biosynthesis and cell proliferation in hyperosmotic environments (Dihazi et al., 2004; Lenassi et al., 2011). The spectrophotometric analysis demonstrated that the content of glycerol was increased by 2.5 fold (Figure 4A) which was consistent with the results of Kogej et al. (2007).

The storage of the compatible sugars and organic acids play a key role for osmotic adjustment of eukaryotic cells (Gagneul et al., 2007; Rosa et al., 2009; Plemenitaš et al., 2014; Sos-Hegedus et al., 2014; Henry et al., 2015). The spectrophotometric analysis demonstrated that the content of compatible sugars was increased by 2.0-fold under salt stress (Figure 4B), suggesting that compatible sugars play a significant role in *A*. *montevidensis* ZYD4 against high salt stress. Further, under salt stress *A*. *montevidensis* ZYD4 showed 1.0- to 2.2-fold increase in the expression of unigenes homologous to hexokinase, 6-phosphofructo-2-kinase, trehalose-phosphate synthase, and mannose-6-phosphate isomerase which participate in the upper pathways of glycolysis, TCA cycle, starch, and sucrose pathway. Nineteen DEGs for the further conversion of glucose-6-phosphate into other intermediates via the down-stream pathway of glycolysis and TCA cycle were suppressed. Unigenes of proline oxidase and pyrroline-5-carboxylate dehydrogenase that generates glutamate via proline cycle increased the expression by 3.6, and 1.9-fold in the salt-induced samples. Moreover, expression level of one unigene encoding glutamate decarboxylase was augmented by 5-fold which was associated with the conversion of glutamate into γ-aminobutyric acid (GABA) suggesting that GABA was accumulated in the salt-induced fungal cells.

## Author contributions

WL, KL, and MX designed research and project outline. KL, XD, and YZ performed growth and morphology observation. KL, XD, MN, and BZ performed transcriptome sample preparation and sequencing. KL, MX, and MN provided the gene functional annotation and differential expression analysis. KL, MN, BL, MX, and WL drafted the manuscript. All authors read and approved the final manuscript.

### Conflict of interest statement

The authors declare that the research was conducted in the absence of any commercial or financial relationships that could be construed as a potential conflict of interest.
