# Supplementary material for: Morphological and Transcriptomic Analysis Reveals the Osmoadaptive Response of Endophytic Fungus Aspergillus montevidensis ZYD4 to High Salt Stress
Source: Front Microbiol. 2017 Sep 21;8:1789. doi: 10.3389/fmicb.2017.01789 (PMC5613514; doi:10.3389/fmicb.2017.01789)
Supplement: Supplementary file 1 [file Table1.docx]

Table S1 Primers used in Q-PCR

Unigenes	Primers (5'to3')
Glyc	TCTATTGTGGTTCCTTGTCG
	CCTTGATCAGGTTATCCTTG
Pro	TTCCATCTCCTCTTCATCC
	GAGTGTGTGCTTAACGAGA
Cys6	CTGCACGGAATGCAGACA
	AGGAGTCGTGAATGACACA
Scf	TCCTCATCAAGAACATGCTC
	GTGCACCATTCAATCACCTT
HMC	CGTTTTGATGCAGCTCTTTG
	GATCCAGTTGATGCTGTTGA
